# Eccentric hamstring strength in young athletes is best documented when normalised to body mass: A cross-sectional study with normative data of 590 athletes from different age categories

**DOI:** 10.5114/biolsport.2023.125585

**Published:** 2023-03-21

**Authors:** Evan Jeanguyot, Benjamin Salcinovic, Amanda Johnson, Nicol Van Dyk, Rod Whiteley

**Affiliations:** 1 NSW Institute of Sport, Sydney, Australia; 2 Aspire Academy Sports Medicine Center, Aspire Academy, Doha, Qatar; 3 Rehabilitation Department, Aspetar Sports Medicine Hospital, Doha, Qatar; 4 Manchester Metropolitan University, Manchester, United Kingdom; 5 Aspetar Orthopaedic and Sports Medicine Hospital, Doha Qatar; 6 High Performance Unit, Irish Rugby Football Union, Dublin, Ireland; 7 Section Sports Medicine, Faculty of Health Sciences, University of Pretoria, Pretoria, South Africa; 8 School of Public Health, Physiotherapy and Sport Sciences, University College Dublin, Dublin, Ireland; 9 School of Human Movement & Nutrition Sciences, The University of Queensland, Australia

**Keywords:** Nordic, Football, Athletics, Strength, Injury, Performance

## Abstract

Despite its widespread use in adults, the Nordic hamstring exercise remains underexplored in athletic youth populations. Further, given the dynamic nature of growth and maturation, comparisons with elite adult populations may be inaccurate. Here we describe absolute and body mass-normalised eccentric hamstring normative values for football, athletics and multi-sport youth populations. 676 routine standardised tests were conducted, including 244 U12–U18 student-athletes (football, athletics, multi-sport) and 346 Qatar Stars League (QSL) football players using the NordBord. The average maximum values for the left and right leg from 3 repetitions were recorded. Significant increases in absolute strength were seen across chronological (e.g., 150 N ± 15 for U12 to 330 N ± 40 for U18) and skeletal (142.9 N ± 13.9 for skeletal age of 12 compared to 336.2 N ± 71.2 for skeletal age of 18) age groups. The differences in values normalised to body mass were smaller at 3.6 N/kg ± 0.25 for the U-13 group, but similar for the U14 to U18 groups (4.5 N/kg ± 0.16, 4.6 N/kg ± 0.11, 4.6 N/kg ± 0.27, 4.7 N/kg ± 0.14, 4.5 N/kg ± 0.18). Students displayed lower absolute strength than the professional football players (272.1 N compared to 297.3 N, p < 0.0001) but higher relative strength (4.7 N/kg compared to 4.2 N/kg, p < 0.0001). Comparing Nordic hamstring strength values between athletes, and between skeletal and chronological age groups can be done when values are normalised to the athlete’s body mass. For the U14s and onwards age categories, body mass normalised values are comparable to professional football players.

## INTRODUCTION

Adolescents undergo a period of rapid growth and development which makes assessment of training-related changes in parameters such as strength difficult to tease out from these normal processes. Between-athlete comparisons are confounded by variations in timing of growth spurts in addition to the longitudinal-within individual differences. Normative eccentric hamstring strength in adult populations has been extensively explored and its association with injury risk mitigation well established [1]. Eccentric hamstring strengthening in youth has been suggested to develop physical qualities of sporting performance as well as reduce injury risk [2]. Further, given that athletic movements for performance in youth mimic their adult counterparts such as deceleration, landing and hopping, exposure to eccentric resistance training is warranted [2]. While its role in injury risk mitigation is well established in adult populations [1], its role in athletic youth populations is not well described. This may in part be due to a lack of normative data for practitioners to base their preparation and intervention decisions on. Understanding any relationships between these strength values and players’ chronological age, and skeletal age (for student athletes) as well as body mass will better inform the interpretation of these tests.

The interactions between growth, maturation, and eccentric hamstring muscle strength in football and athletic youth populations are yet to be fully established. Further, a comprehensive overview assessing eccentric hamstring strength between elite footballers and elite youth athletes is yet to be explored. The inherent complexity and non-linearity of youth athletes’ normal physical growth, biological maturation, and behavioural development make it difficult to gauge a true representation of their abilities at any given moment [3]. These complex interactions need to be considered when assessing eccentric hamstring strength at a single time point, or when comparing different measures over time. To better understand the effectiveness of training interventions, any improvements in strength which are attributable to these training effects need to be teased out from those due to physical growth and maturation. [1, 2]

This paper aims to describe normative values of eccentric hamstring strength in well-trained male athletic and football youth populations. Secondly, it aims to describe any relationships between eccentric hamstring strength and body mass, skeletal maturation status, and chronological age. Finally, these data are compared with a sample of professional adult male football players.

## MATERIALS AND METHODS

### Study design and participants

#### Student athletes

**FIG. 1:**
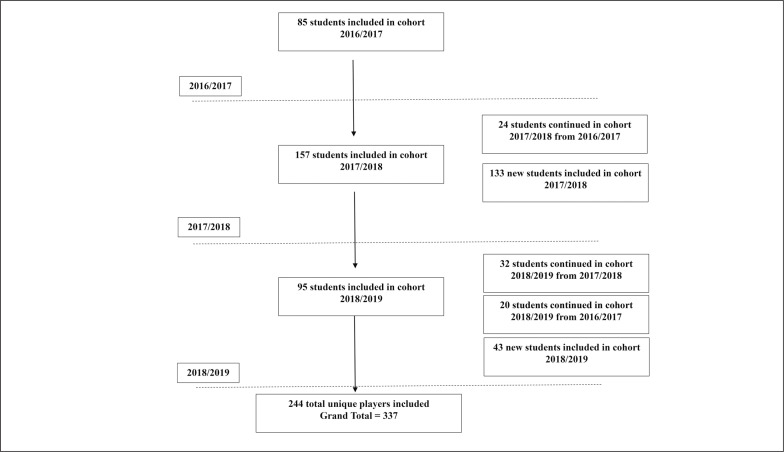
Flow chart demonstrating the movement of players and repeated measurements during three seasons, 2016–17 to 2018–19 and body mass (kg) for highly trained athletes in sports academy.

A cross-sectional cohort study design described growth, maturation, and knee flexor strength prospectively over three seasons. The participants were male full-time student athletes, enrolled in the football, athletics or multi-sports programmes at Aspire Academy, an elite sporting academy in the Doha, Qatar. Testing was completed during the preseason or initial competitive cycle of the 2016/17, 2017/18 and 2018/19 seasons (Figure 1).

A total of 330 male athletes (chronological mean age 15.3 ± 1.7, height 169 ± 11, weight 58.8 ± 14.2, BMI 20.3 ± 3.3) presented for screening across three seasons from 2016 to 2019. The demographic data for this cohort are summarised in Table 1. There were 313 body mass measurements and 306 complete skeletal age measures conducted at the time of eccentric hamstring testing assessments. All athletes needed to be free of injury to the lower limbs and able to participate fully in training at the time of testing.

**TABLE 1:** Participant characteristics. Student athletes compete in age categories “U13” to “U20” – Under 13 years of age at the start of the academic year to under 20 years of age respectively. Of these student athletes, 174 played football, 119 competed in athletics, and 46 in multi-sport events. The QSL Adult cohort is the professional football players included in pre-competition medical assessment. Data are presented as mean ± SD. Skeletal age is assessed using the FELS method. No skeletal age data are available for the adult QSL athletes.

Age Group	Chronological Age (Years)	Skeletal Age (Years)	Height (cm)	Weight (kg)	Body Mass Index (kg/m²)
**Student athletes (n = 330)**	15.2 ± 1.7	16.1 ± 2.0	168.7 ± 10.4	58.0 ± 13.6	20.1 ± 3.3

U13 (n = 38)	12.6 ± 0.46	13.1 ± 1.3	156.7 ± 7.8	46.4 ± 13.6	18.7 ± 3.7
U14 (n = 61)	13.5 ± 0.4	14.4 ± 1.1	160.0 ± 8.4	47.9 ± 11.1	18.5 ± 3.0
U15 (n = 81)	14.9 ± 0.7	15.9 ± 1.6	169.4 ± 8.9	58.3 ± 11.3	20.2 ± 3.2
U16 (n = 31)	15.4 ± 0.4	16.7 ± 1.4	174.1 ± 7.6	58.8 ± 8.7	19.3 ± 1.9
U17 (n = 67)	16.5 ± 0.4	17.6 ± 0.8	174.3 ± 6.0	65.3 ± 8.9	21.5 ± 2.6
U18 (n = 52)	17.5 ± 0.4	17.8 ± 0.8	175.7 ± 8.5	67.6 ± 13.5	21.8 ± 3.3

**QSL football (n = 346)**	25.9 ± 4.8	N/A	176.6 ± 6.9	72.3 ± 9.3	23.1 ± 2.1

#### Qatar Stars League footballers

The comparison adult data are drawn from a previously published study [4] which prospectively examined professional footballers in the Qatar Stars’ League over a 4-year period during annual pre-competition medical assessment. In year 2 of this study, all eligible players were tested on the NordBord Nordic hamstring testing device. After exclusion of the players who were injured or refused to consent to the testing, 346 players’ results were available for this analysis.

### Data collection procedures and statistics

#### Eccentric knee flexor strength

**FIG. 2:**
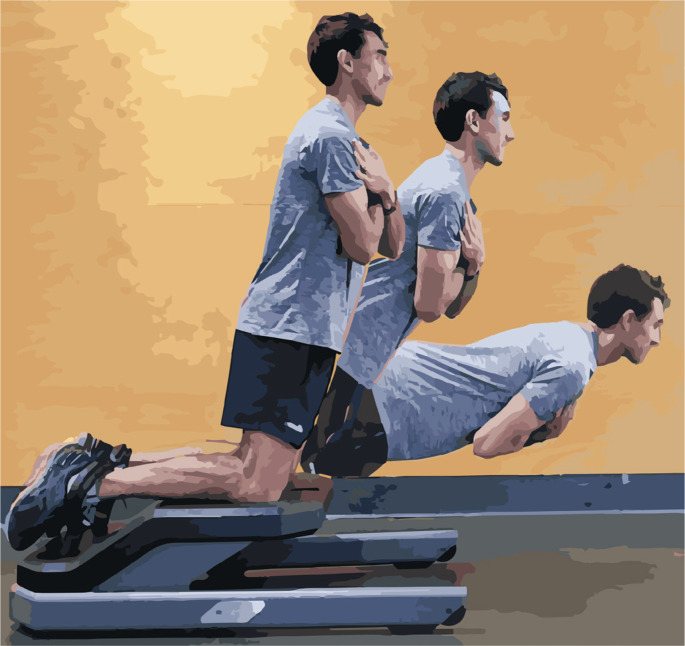
Nordic hamstring exercise being performed on the NordBord and body mass (kg) for highly trained athletes in sports academy.

Athletes performed one set of three maximal repetitions on a device specifically designed to measure maximal force output (N) during the Nordic hamstring exercise [5] using previously described methods. The NordBord (Vald Performance, Australia) has been previously shown to have moderate to high reliability (intraclass correlation coefficient = 0.83–0.90; typical error, 21.7–27.5 N; typical error as a coefficient of variation, 5.8%-8.5%) [5]. Briefly, the athletes were first shown an instructional video and provided a handout in English and Arabic explaining the correct technique during the exercise. Each athlete was positioned kneeling on the device, the ankles secured by individual ankle braces immediately above the lateral malleoli. A submaximal effort was performed for familiarisation and to ensure correct performance. Participants were instructed to gradually lean forward at the slowest possible speed while maximally resisting the fall with both legs and maintaining an upright posture with their spine and pelvis in a neutral position. As the athlete lowered to the ground, uniaxial load cells attached to the ankle braces (Delphi Measurement, Gold Coast, Australia) measured the concomitant maximal force output (N). Each repetition was characterised by a distinct peak in maximum pull force followed by a sharp decline. This rapid reduction in force indicated completion of a repetition, whereby the athlete fails to maintain the resistance required to eccentrically lower their trunk position against the increasing demands of body mass, gravity, and the distance from the line of pull. Verbal encouragement was provided throughout the exercise to ensure maximal effort. The proprietary software provided instantaneous raw data that were then exported into a customised Microsoft Excel spreadsheet (Microsoft, Redmond, USA). The mean of the left and right maximum force (N) was taken to determine a between-limb absolute maximum strength score. An illustrated version of the Nordic hamstring exercise can be seen in Figure 2.

#### Skeletal bone age and anthropometry

Skeletal maturation was assessed at the beginning of each academic year, using X-ray images of the athlete’s left hand and wrist complex taken at the Radiology Department at Aspetar Orthopaedic and Sports Medicine Hospital. The images were interpreted and entered into an academy maturation database by the same experienced assessor. Skeletal age was determined using the Fels method, following the procedures outlined by Roche et al. [6]; here a maximal skeletal age of 18.0 indicates full maturity. Anthropometric screenings were conducted by ISAK (International Society for the Advancement of Kinanthropometry) Level 2 certified academy staff at the start and end of each season, which corresponded to the academic year. Measures were taken early in the morning prior to any activities to minimize diurnal variations, following ISAK-recommended procedures, and were uploaded to a central academy anthropometry database, following the procedures previously described [7].

### Data analysis

Absolute force data for the left and right limbs were entered in a spreadsheet (Microsoft Excel Microsoft, Redmond, USA) which calculated the average of both legs for the repetition where the highest force was displayed for each leg during the three contractions – the “mean peak force” (average of both limbs’ highest score). This value was recorded in both absolute terms (N) and relative to body mass (N/kg). Statistical analysis was conducted in JMP (JMP, Version 16.0 SAS Institute Inc., Cary, NC, 1989–2019). Mixed model regression analysis was conducted considering the fixed effects of sport (Football, Athletics, and Multi-sport) with chronological age group (U13 to U18), and the interaction effects of these two factors for the student athletes (considered as random effects), with post hoc adjustment for multiple comparison (Tukey’s HSD).

### Ethical approval and consent

This study was part of a larger study on growth, maturation and athletic development for which written informed consent was obtained from the athletes’ guardians prior to data collection and ethics approval was granted from the Anti-Doping Lab Qatar Institutional Review Board (IRB Application #E20140000012). The adult data were collected as part of the routine pre-participation periodic health evaluation for football players participating in the Qatar Stars’ League and ethical approval for this cohort was obtained from the Shafallah Medical Genetics Centre (institutional review board project number 2012–020).

## RESULTS

### Student athlete Nordic hamstring strength – absolute and relative values for different age group categories and sports

These analyses were conducted for both absolute and relative Nordic hamstring strength. For absolute strength, a significant effect of age group was found (p < 0.001) and a non-significant effect of sport (p > 0.20). The interaction effect (sport and age group) was significant (p < 0.01). For the Nordic hamstring strength normalised to body mass, there was a significant effect of both chronological age (p = 0.004) and an interaction effect of sport and chronological age (p = 0.0014) whereas the effect of sport was not significant (p = 0.7112). Post hoc testing however revealed that of the possible 210 pair-wise comparisons (age group and sport), 21 were statistically significant after adjusting for multiple comparison (Tukey’s HSD), Appendix 5, Figure 4. Post hoc multiple comparison of the different student athlete age group categories for the relative Nordic hamstring strength revealed significant differences only for the comparison between the U13 years age group and the 15 years (difference = -1.0 N/kg [-1.7 to -0.2] p = 0.005), and the U13 to the U17 years age groups (difference = -1.1 N/kg [-1.9 to -0.3] p = 0.002). For each of these models, the residuals were analysed for normality by a combination of inspection of frequency histograms and residual quantile-quantile plots, and Shapiro-Wilk tests.

### Nordic strength compared to chronological and skeletal age

**FIG. 3:**
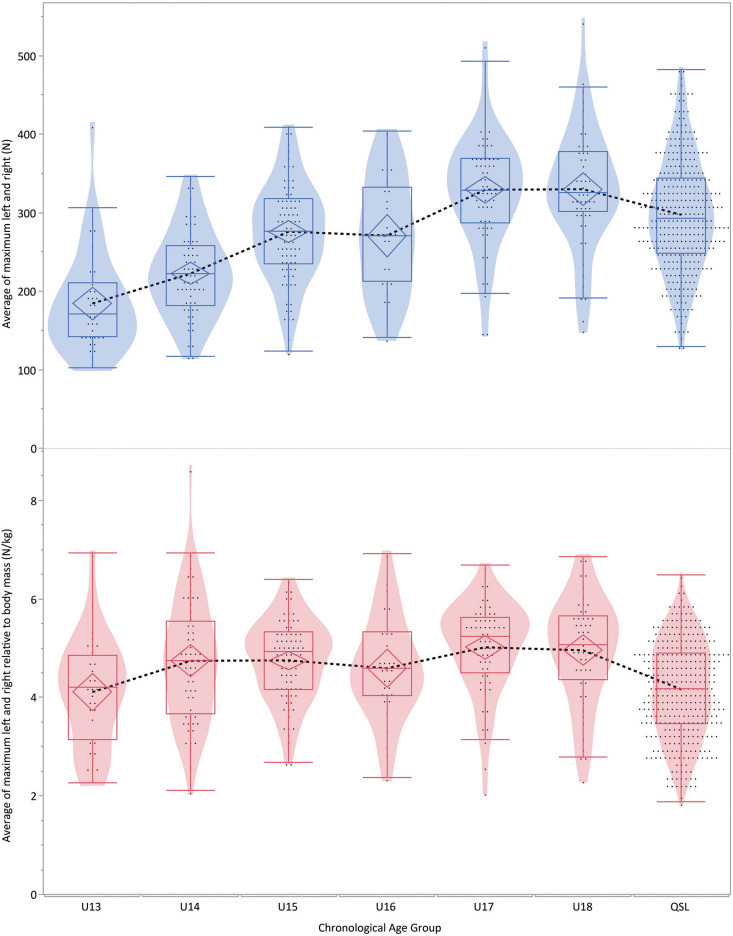
Relative (N/kg) and absolute strength values for the different age groups. Note that the “QSL” category indicates all the Qatar Stars’ League players, and the U13 to U20 categories indicate the Aspire Academy student athletes’ age group categories. Confidence diamond within the boxplot describes the 95% confidence limit of the mean, contours represent the distribution of the individual points (dots) for each observation. Dotted line connects the mean values of the groups.

Figure 3 and Table 2 show the absolute and relative values of Nordic hamstring strength for the different age and sport categories respectively. Comparing the adult (Qatar Stars’ League players) to the student athlete age groups, significant differences were found for absolute strength with the U13, U14, U17, and U18 age categories (Figure 3, Appendix 1). By contrast, comparison of the normalised Nordic hamstring strength only showed significant differences for the 13 years age group compared to the 15 and 17 years’ categories (Figure 3, Appendix 2).

**TABLE 2:**
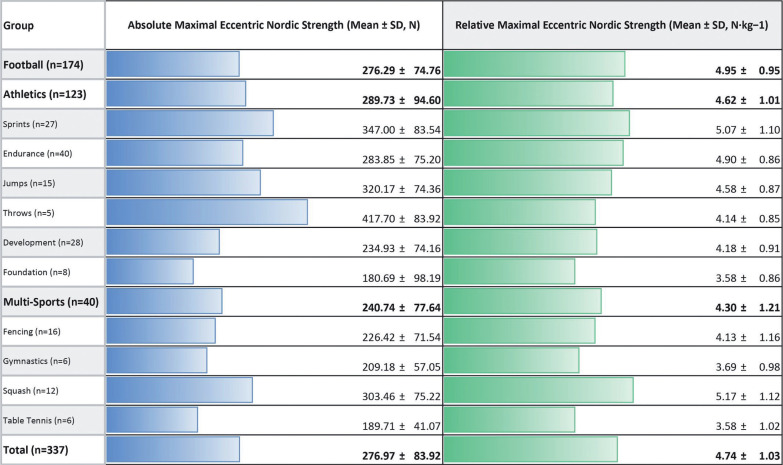
Absolute and relative strength values of the Nordic hamstring exercise by sports category

**FIG. 4:**
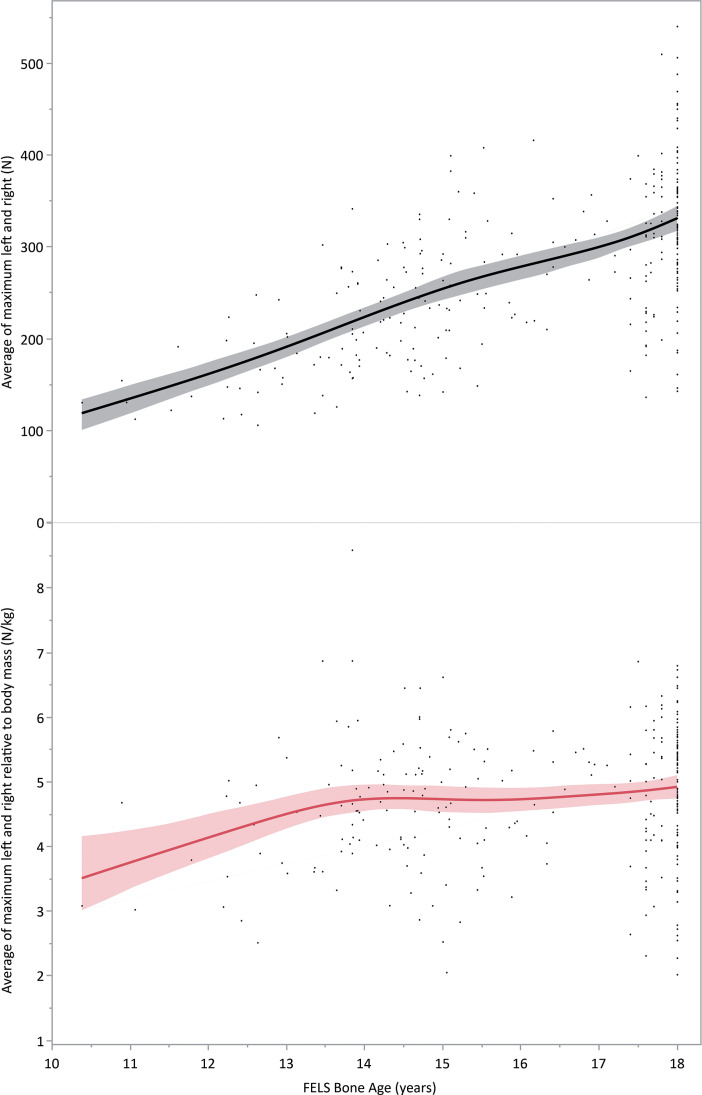
Absolute and relative Nordic hamstring strength values compared to skeletal age as estimated by the Fels method. There is a moderate positive (r = 0.65 [0.57 to 0.71]) significant (p < 0.0001) correlation between Fels skeletal age and absolute (N) strength, but only a weak positive (r = 0.18 [0.06 to 0.29]) significant (p = 0.003) correlation between relative strength (N/kg) and skeletal age.

**FIG. 5:**
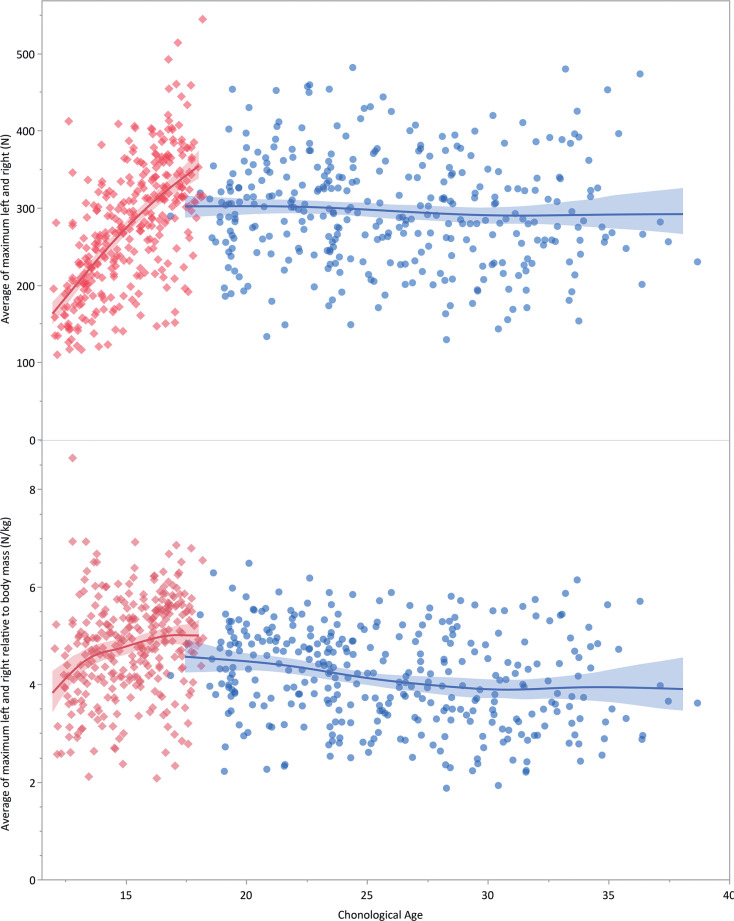
Absolute (N, upper panel) and relative (N/kg, lower panel) average maximum Nordic hamstring strength compared to age for both the student athletes (red diamonds, red line of best fit with confidence interval) and the QSL football players (blue circles, blue line). There is a moderate positive (r = 0.64 [0.57 to 0.70]) significant (p < 0.0001) correlation of absolute strength and age for the student athletes but not for the QSL footballers (r = 0.06 [-0.17 to 0.04], p = 0.2365). For the relative strengths, there is a weak positive (r = 0.25 [0.14 to 0.36]) significant (p < 0.0001) correlation for the student athletes, and a weak negative (r = -0.21 [-0.31 to -0.11)] significant (p < 0.0001) correlation for the QSL football players.

Figures 4 and 5 show these same absolute and relative Nordic strength values compared to skeletal age and chronological age for the student athletes and QSL football players respectively.

## DISCUSSION

This cross-sectional study of highly trained male youth athletes showed a clear pattern of increasing strength with chronological and skeletal age. However, the more important clinical finding is the more consistent relationship between body mass and maximum eccentric Nordic strength across these chronological and skeletal ages, particularly once these athletes were above the age of 13. This information can be used by physical preparation and rehabilitation practitioners as benchmarks for their adolescent male cohorts independent of their size and age. While this was a cross-sectional study, the stability of this finding across ages suggests that longitudinal progress needs to be assessed using the body mass-normalised values lest the apparent maturation effect be mistaken for relative improvement in strength.

These data suggest that clinicians should consider body mass as a critical factor in the interpretation of absolute eccentric hamstring strength. Increased body mass and/or longer lower leg lever distances can influence eccentric hamstring strength results during the Nordic exercise [8]. Specifically, heavier and older players have been shown to outperform their younger, lighter counterparts [8]. Body mass was found to be largely responsible for observed age-related increases in absolute Nordic hamstring strength [9]. A large body of evidence suggests that performing the Nordic hamstring exercise as part of a prevention programme is an effective way to reduce this injury’s burden [1]. These current data may complement this knowledge by providing practitioners with strength training targets for those adolescents beginning resistance training.

The student athletes’ average normative score was 4.74 N/kg (95% confidence interval: 4.64 to 4.86) whereas the QSL players’ average was 4.16 N/kg (4.06 to 4.27). Soccer players have been shown to achieve eccentric hamstring strength (N) scores of 4x body mass (kg) +26.1 N; for example a 50 kg athlete would have a predictive score of: 4 × 50 kg +26.1 = 226.1 N [8]. This value is comparable with the mean absolute strength of 277 N described in the current research. Roe et al. [10] examined eccentric knee flexor strength profiles of 341 elite Gaelic football players, 105 of whom were U17 or below, and found body mass to have moderate-to-large associations (r = 0.47) with maximum force in youth. In this study, relative maximum force was found to be 4.4 N/kg across all players. Bourne et al. [11] reported eccentric knee flexor strength in elite rugby union players of 3.65 ± 0.71 N/kg [11]. Further, they found subelite and U19 players to be significantly stronger than elite players, which is consistent with our cohort. Again, in uninjured elite Australian footballers, in-season relative eccentric hamstring strength scores were found to be 4.09 ± 1.01 N/kg [12]. Together these findings suggest that long-term tracking of these body mass normalised Nordic values can begin in early adolescence – we suggest after the age of 13 – and progress throughout an athlete’s career to adulthood; however, this would require verification with longitudinal studies.

Eccentric hamstring strength training in youth has been advocated as important in developing physical qualities that underpin performance and reduce injury risk [2, 9]. Furthermore, muscular strength in youth is strongly associated with a multitude of physical qualities including running speed, power, agility and endurance performance [13] Eccentric hamstring strength training complements the Youth Physical Development model to facilitate appropriate neural and structural adaptations [14, 15]. Despite the clinical indication for this exercise, performance and rehabilitation staff must consider the unique journey each athlete follows. It is important to recognise and embrace the inherent complexity and non-linearity of athlete development rather than age-related prescription-based methods [3]. The Nordic hamstring exercise in male youth footballers has been established as a reliable measure of bilateral hamstring peak force across maturation stages [16]. Individual differences in growth and maturation may contribute to competitive inequity and increased risk of injury [17]. Our findings highlight the fact that there are moderate associations between skeletal age and chronological age with eccentric hamstring strength in absolute terms but not when body mass is accounted for. Similarly, non-linear relationships between Nordic strength and age have been found in highly trained youth footballers [9]. Interestingly, abrupt changes were found in the U16 age group in absolute terms. This was ascribed to the pubertal growth spurt and accompanying increase in serum androgen hormones [9]. Drury et al. (2019) explored the influence of maturation status on eccentric hamstring strength improvements by implementing a 6-week Nordic hamstring exercise intervention study. Small and moderate increases (10% and 16%) in relative eccentric hamstring strength were observed in the pre-peak height velocity (PHV) and mid/post PHV groups respectively. Based on these findings, the authors suggest ingraining relative strength as a foundation for absolute strength in less mature individuals [2]. This considered, appropriate training relative to a child’s chosen activity, before and during maturation, enables combinatory and consolidatory factors that support motor skill performance during post-pubertal training years driven primarily via increases in testosterone, growth hormone and insulin-like growth factor [18]. Future research may consider the effect of hormonal as well as physical (i.e. body size) changes to tease out any individual effects.

### Limitations and future research

Given the limited understanding of eccentric hamstring strength in youth sporting populations, further exploration is warranted particularly in other cohorts including youth female athletes and other ethnicities. In this cohort, the lever arm was not measured and may represent an extra independent variable to consider in youth athletes. The Nordic hamstring exercise requires a level of technical competence and a capacity to tolerate a high loading stimulus, which limits these findings to athletes who have been given appropriate technical instruction. The cross-sectional nature of this study prevents definitive statements about the longitudinal stability of these measures during maturation irrespective of the apparent stability of these data. Accordingly, long-term data examining individual variation are suggested for future research. Finally, while the performance of the Nordic hamstring exercise is associated with a reduction in hamstring injury in adults, there are essentially no similar data for adolescent athletes. Recommending the measurement of this strength in the absence of such data must be considered preliminary until this research is conducted.

## CONCLUSIONS

Adolescent male athletes show increases in the absolute strength displayed during the Nordic hamstring exercise as chronological and especially skeletal age increases; however, when normalised to body mass these strengths are quite stable at approximately 4.7 N/kg once these adolescent boys were over the age of 13 years.

## APPENDIX 1 All pairwise comparisons of the different age groups for the absolute values of Nordic hamstring strength showing the mean, standard error of the difference, and the lower and upper 95% confidence limits. Significant (p < 0.05) differences are highlighted in red font (post hoc adjustment for multiple comparison using Tukey’s HSD)

**Tukey HSD All Pairwise Comparisons**

Quantile = 2.86867, Adjusted DF = 293.2, Adjustment = Tukey-Kramer

**All Pairwise Differences**

**Table:**

Chronological Age Group	Chronological Age Group	Difference	Std Error	t Ratio	Prob > |t|	Lower 95%	Upper 95%
U13	U14	-39.812	19.88504	-2.00	0.3436	-96.856	17.2314
U13	U15	-92.807	18.79877	-4.94	< .0001*	-146.734	-38.8792
U13	U16	-109.499	22.59550	-4.85	< .0001*	-174.318	-44.6797
U13	U17	-143.100	19.20479	-7.45	< .0001*	-198.192	-88.0076
U13	U18	-152.554	21.16543	-7.21	< .0001*	-213.271	-91.8375
U14	U15	-52.994	12.21499	-4.34	0.0003*	-88.035	-17.9536
U14	U16	-69.686	17.92088	-3.89	0.0017*	-121.096	-18.2774
U14	U17	-103.288	13.51347	-7.64	< .0001*	-142.053	-64.5219
U14	U18	-112.742	16.18616	-6.97	< .0001*	-159.175	-66.3091
U15	U16	-16.692	15.69550	-1.06	0.8954	-61.717	28.3332
U15	U17	-50.293	11.08316	-4.54	0.0001*	-82.087	-18.4992
U15	U18	-59.747	14.33989	-4.17	0.0006*	-100.884	-18.6110
U16	U17	-33.601	15.97462	-2.10	0.2884	-79.427	12.2249
U16	U18	-43.055	19.03310	-2.26	0.2131	-97.655	11.5443
U17	U18	-9.454	14.40274	-0.66	0.9864	-50.771	31.8624

## APPENDIX 2 All pairwise comparisons of the different age groups for the relative values of Nordic hamstring strength showing the mean, standard error of the difference, and the lower and upper 95% confidence limits. Significant (p < 0.05) differences are highlighted in red font (post hoc adjustment for multiple comparison using Tukey’s HSD)

**Tukey HSD All Pairwise Comparisons**

Quantile = 2.86946, Adjusted DF = 281.5, Adjustment = Tukey-Kramer

**All Pairwise Differences**

**Table:**

Chronological Age Group	Chronological Age Group	Difference	Std Error	t Ratio	Prob > |t|	Lower 95%	Upper 95%
U13	U14	-0.83261	0.2953740	-2.82	0.0574	-1.68018	0.01495
U13	U15	-1.00024	0.2781175	-3.60	0.0051*	-1.79828	-0.20219
U13	U16	-1.01200	0.3695513	-2.74	0.0710	-2.07241	0.04841
U13	U17	-1.10295	0.2864349	-3.85	0.0020*	-1.92487	-0.28104
U13	U18	-0.84518	0.3121202	-2.71	0.0768	-1.74080	0.05044
U14	U15	-0.16763	0.1903528	-0.88	0.9509	-0.71384	0.37858
U14	U16	-0.17939	0.3119213	-0.58	0.9926	-1.07444	0.71566
U14	U17	-0.27034	0.2072105	-1.30	0.7823	-0.86493	0.32424
U14	U18	-0.01257	0.2413914	-0.05	1.0000	-0.70523	0.68010
U15	U16	-0.01176	0.2884429	-0.04	1.0000	-0.83944	0.81591
U15	U17	-0.10272	0.1753471	-0.59	0.9919	-0.60587	0.40043
U15	U18	0.15506	0.2157032	0.72	0.9795	-0.46389	0.77401
U16	U17	-0.09095	0.2947299	-0.31	0.9996	-0.93667	0.75476
U16	U18	0.16682	0.3257376	0.51	0.9957	-0.76787	1.10151
U17	U18	0.25778	0.2223961	1.16	0.8558	-0.38038	0.89593

## APPENDIX 3 All pairwise comparisons for the different skeletal age groups for the student athletes for absolute Nordic hamstring strength. Significant differences are noted in the column “Prob > |t|” with an asterisk and a coloured font.

**Table:**

FELS Bone Age Group	FELS Bone Age Group	Difference	Std Error	t Ratio	Prob > |t|	Lower 95%	Upper 95%
10	11	-2.188	49.29283	-0.04	1.0000	-156.243	151.868
10	12	-29.533	40.81834	-0.72	0.9984	-157.103	98.037
10	13	-64.229	39.08058	-1.64	0.7799	-186.368	57.910
10	14	-96.338	38.51122	-2.50	0.2355	-216.698	24.021
10	15	-129.486	38.82598	-3.34	0.0265*	-250.829	-8.142
10	16	-157.938	41.06059	-3.85	0.0046*	-286.265	-29.610
10	17	-160.318	38.26461	-4.19	0.0012*	-279.907	-40.729
10	18	-193.303	37.81667	-5.11	< .0001*	-311.491	-75.114
11	12	-27.346	36.31838	-0.75	0.9979	-140.852	86.160
11	13	-62.042	34.35375	-1.81	0.6783	-169.408	45.324
11	14	-94.151	33.70463	-2.79	0.1218	-199.488	11.187
11	15	-127.298	34.06384	-3.74	0.0068*	-233.758	-20.838
11	16	-155.750	36.59043	-4.26	0.0009*	-270.106	-41.394
11	17	-158.131	33.42258	-4.73	0.0001*	-262.587	-53.675
11	18	-191.115	32.90880	-5.81	< .0001*	-293.965	-88.265
12	13	-34.696	20.40917	-1.70	0.7462	-98.481	29.089
12	14	-66.805	19.29654	-3.46	0.0176*	-127.112	-6.497
12	15	-99.952	19.91731	-5.02	< .0001*	-162.200	-37.705
12	16	-128.404	23.98362	-5.35	< .0001*	-203.360	-53.448
12	17	-130.785	18.79955	-6.96	< .0001*	-189.539	-72.030
12	18	-163.769	17.87017	-9.16	< .0001*	-219.619	-107.919
13	14	-32.109	15.28109	-2.10	0.4745	-79.867	15.649
13	15	-65.257	16.05785	-4.06	0.0020*	-115.442	-15.071
13	16	-93.708	20.88945	-4.49	0.0004*	-158.994	-28.422
13	17	-96.089	14.64849	-6.56	< .0001*	-141.870	-50.308
13	18	-129.073	13.43496	-9.61	< .0001*	-171.062	-87.085
14	15	-33.148	14.61768	-2.27	0.3654	-78.832	12.537
14	16	-61.599	19.80382	-3.11	0.0522	-123.493	0.294
14	17	-63.980	13.05375	-4.90	< .0001*	-104.777	-23.183
14	18	-96.964	11.67563	-8.30	< .0001*	-133.454	-60.474
15	16	-28.452	20.40917	-1.39	0.8995	-92.237	35.333
15	17	-30.832	13.95504	-2.21	0.4023	-74.446	12.781
15	18	-63.817	12.67530	-5.03	< .0001*	-103.431	-24.203
16	17	-2.381	19.31988	-0.12	1.0000	-62.761	58.000
16	18	-35.365	18.41678	-1.92	0.6003	-92.923	22.193
17	18	-32.984	10.83452	-3.04	0.0629	-66.846	0.877

## APPENDIX 4 All pairwise comparisons of relative Nordic hamstring strength for the different skeletal age groups. No significant differences were found for any between-group comparisons.

**Table:**

FELS Bone Age Group	FELS Bone Age Group	Difference	Std Error	t Ratio	Prob > |t|	Lower 95%	Upper 95%
10	11	0.47335	0.9687062	0.49	0.9999	-2.55567	3.502366
10	12	-0.20649	0.7398616	-0.28	1.0000	-2.51994	2.106965
10	13	-0.76756	0.7074430	-1.08	0.9760	-2.97965	1.444518
10	14	-0.82032	0.7007274	-1.17	0.9620	-3.01140	1.370768
10	15	-0.60721	0.7054303	-0.86	0.9947	-2.81300	1.598585
10	16	-1.06531	0.7322731	-1.45	0.8751	-3.35503	1.224416
10	17	-0.86670	0.6980271	-1.24	0.9464	-3.04934	1.315940
10	18	-0.99327	0.6922274	-1.43	0.8834	-3.15777	1.171239
11	12	-0.67983	0.7398616	-0.92	0.9918	-2.99328	1.633619
11	13	-1.24091	0.7074430	-1.75	0.7122	-3.45299	0.971173
11	14	-1.29366	0.7007274	-1.85	0.6512	-3.48475	0.897422
11	15	-1.08055	0.7054303	-1.53	0.8397	-3.28634	1.125239
11	16	-1.53865	0.7322731	-2.10	0.4747	-3.82838	0.751070
11	17	-1.34005	0.6980271	-1.92	0.6007	-3.52269	0.842595
11	18	-1.46661	0.6922274	-2.12	0.4628	-3.63112	0.697893
12	13	-0.56108	0.3308762	-1.70	0.7488	-1.59569	0.473529
12	14	-0.61383	0.3162631	-1.94	0.5860	-1.60274	0.375084
12	15	-0.40072	0.3265508	-1.23	0.9499	-1.42180	0.620364
12	16	-0.85882	0.3810870	-2.25	0.3743	-2.05043	0.332789
12	17	-0.66021	0.3102343	-2.13	0.4564	-1.63028	0.309849
12	18	-0.78678	0.2969549	-2.65	0.1715	-1.71532	0.141759
13	14	-0.05275	0.2304404	-0.23	1.0000	-0.77331	0.667806
13	15	0.16036	0.2443682	0.66	0.9992	-0.60375	0.924468
13	16	-0.29774	0.3135406	-0.95	0.9898	-1.27814	0.682658
13	17	-0.09914	0.2220939	-0.45	1.0000	-0.79359	0.595323
13	18	-0.22570	0.2031320	-1.11	0.9722	-0.86087	0.409465
14	15	0.21311	0.2241856	0.95	0.9897	-0.48789	0.914111
14	16	-0.24499	0.2980789	-0.82	0.9961	-1.17705	0.687063
14	17	-0.04638	0.1996724	-0.23	1.0000	-0.67073	0.577966
14	18	-0.17295	0.1783422	-0.97	0.9883	-0.73060	0.384703
15	16	-0.45810	0.3089726	-1.48	0.8628	-1.42422	0.508015
15	17	-0.25950	0.2155971	-1.20	0.9552	-0.93364	0.414649
15	18	-0.38606	0.1960077	-1.97	0.5659	-0.99895	0.226829
16	17	0.19861	0.2916744	0.68	0.9990	-0.71342	1.110636
16	18	0.07204	0.2775083	0.26	1.0000	-0.79569	0.939774
17	18	-0.12657	0.1674183	-0.76	0.9979	-0.65006	0.396929

## APPENDIX 5 All pairwise comparisons of relative Nordic hamstring strength for the student athletes – age and sport, with post-hoc adjustment (Tukey HSD) for multiple comparison. Significant effects are highlighted in the Prob > |t| column with an asterisk and coloured font.

**Tukey HSD All Pairwise Comparisons**

Quantile = 3.52198, Adjusted DF = 278.9, Adjustment = Tukey-Kramer

**All Pairwise Differences**

**Table:**

SPORT 2	Chronological Age Group	SPORT 2	Chronological Age Group	Difference	Std Error	t Ratio	Prob > |t|	Lower 95%	Upper 95%
Athletics	U13	Athletics	U14	-0.01462	0.4174617	-0.04	1.0000	-1.48491	1.45568
Athletics	U13	Athletics	U15	-0.69500	0.3555572	-1.95	0.8876	-1.94727	0.55726
Athletics	U13	Athletics	U16	-0.67331	0.3574688	-1.88	0.9156	-1.93231	0.58569
Athletics	U13	Athletics	U17	-1.36840	0.3529221	-3.88	0.0153*	-2.61139	-0.12542
Athletics	U13	Athletics	U18	-1.54723	0.3693259	-4.19	0.0048*	-2.84799	-0.24647
Athletics	U13	Football	U13	-0.83713	0.3607736	-2.32	0.6678	-2.10777	0.43351
Athletics	U13	Football	U14	-1.18520	0.3344155	-3.54	0.0467*	-2.36300	-0.00739
Athletics	U13	Football	U15	-1.27168	0.3332591	-3.82	0.0190*	-2.44541	-0.09794
Athletics	U13	Football	U16	-0.41366	0.7029482	-0.59	1.0000	-2.88943	2.06211
Athletics	U13	Football	U17	-1.38900	0.3461831	-4.01	0.0094*	-2.60825	-0.16975
Athletics	U13	Football	U18	-1.30046	0.3497937	-3.72	0.0265*	-2.53243	-0.06849
Athletics	U13	Multi-sport	U13	1.09518	0.7296358	1.50	0.9900	-1.47459	3.66494
Athletics	U13	Multi-sport	U14	-1.03998	0.4496943	-2.31	0.6735	-2.62379	0.54384
Athletics	U13	Multi-sport	U15	-0.77599	0.3889837	-1.99	0.8694	-2.14598	0.59400
Athletics	U13	Multi-sport	U16	-1.69099	0.5473588	-3.09	0.1676	-3.61878	0.23680
Athletics	U13	Multi-sport	U17	-0.29342	0.4317714	-0.68	1.0000	-1.81411	1.22728
Athletics	U13	Multi-sport	U18	0.57020	0.5572685	1.02	0.9999	-1.39249	2.53289
Athletics	U14	Athletics	U15	-0.68039	0.3346714	-2.03	0.8506	-1.85909	0.49832
Athletics	U14	Athletics	U16	-0.65869	0.3512654	-1.88	0.9186	-1.89584	0.57846
Athletics	U14	Athletics	U17	-1.35379	0.3482578	-3.89	0.0148*	-2.58034	-0.12723
Athletics	U14	Athletics	U18	-1.53262	0.3649971	-4.20	0.0046*	-2.81813	-0.24710
Athletics	U14	Football	U13	-0.82252	0.3563366	-2.31	0.6767	-2.07753	0.43249
Athletics	U14	Football	U14	-1.17058	0.3296238	-3.55	0.0456*	-2.33151	-0.00965
Athletics	U14	Football	U15	-1.25706	0.3284506	-3.83	0.0182*	-2.41386	-0.10026
Athletics	U14	Football	U16	-0.39904	0.7006813	-0.57	1.0000	-2.86683	2.06875
Athletics	U14	Football	U17	-1.37439	0.3415566	-4.02	0.0090*	-2.57734	-0.17143
Athletics	U14	Football	U18	-1.28584	0.3452156	-3.72	0.0259*	-2.50169	-0.07000
Athletics	U14	Multi-sport	U13	1.10979	0.7274521	1.53	0.9881	-1.45228	3.67187
Athletics	U14	Multi-sport	U14	-1.02536	0.4461425	-2.30	0.6839	-2.59667	0.54594
Athletics	U14	Multi-sport	U15	-0.76137	0.3848720	-1.98	0.8771	-2.11689	0.59414
Athletics	U14	Multi-sport	U16	-1.67637	0.5444445	-3.08	0.1719	-3.59390	0.24115
Athletics	U14	Multi-sport	U17	-0.27880	0.4280709	-0.65	1.0000	-1.78646	1.22886
Athletics	U14	Multi-sport	U18	0.58482	0.5544063	1.05	0.9999	-1.36779	2.53743
Athletics	U15	Athletics	U16	0.02169	0.2584407	0.08	1.0000	-0.88853	0.93192
Athletics	U15	Athletics	U17	-0.67340	0.2691777	-2.50	0.5311	-1.62144	0.27464
Athletics	U15	Athletics	U18	-0.85223	0.2919248	-2.92	0.2489	-1.88039	0.17592
Athletics	U15	Football	U13	-0.14213	0.2812871	-0.51	1.0000	-1.13282	0.84856
Athletics	U15	Football	U14	-0.49019	0.2465735	-1.99	0.8726	-1.35862	0.37823
Athletics	U15	Football	U15	-0.57667	0.2450029	-2.35	0.6432	-1.43957	0.28622
Athletics	U15	Football	U16	0.28134	0.6656583	0.42	1.0000	-2.06309	2.62578
Athletics	U15	Football	U17	-0.69400	0.2623119	-2.65	0.4239	-1.61786	0.22986
Athletics	U15	Football	U18	-0.60546	0.2670588	-2.27	0.7061	-1.54603	0.33512
Athletics	U15	Multi-sport	U13	1.79018	0.6937818	2.58	0.4718	-0.65331	4.23367
Athletics	U15	Multi-sport	U14	-0.34498	0.3888313	-0.89	1.0000	-1.71443	1.02448
Athletics	U15	Multi-sport	U15	-0.08099	0.3166593	-0.26	1.0000	-1.19626	1.03428
Athletics	U15	Multi-sport	U16	-0.99599	0.4985644	-2.00	0.8680	-2.75192	0.75995
Athletics	U15	Multi-sport	U17	0.40159	0.3679556	1.09	0.9998	-0.89435	1.69752
Athletics	U15	Multi-sport	U18	1.26520	0.5094242	2.48	0.5449	-0.52898	3.05939
Athletics	U16	Athletics	U17	-0.69509	0.2547918	-2.73	0.3662	-1.59246	0.20228
Athletics	U16	Athletics	U18	-0.87392	0.2912006	-3.00	0.2070	-1.89953	0.15168
Athletics	U16	Football	U13	-0.16382	0.2836996	-0.58	1.0000	-1.16301	0.83536
Athletics	U16	Football	U14	-0.51189	0.2493221	-2.05	0.8401	-1.39000	0.36622
Athletics	U16	Football	U15	-0.59837	0.2477690	-2.42	0.5971	-1.47100	0.27427
Athletics	U16	Football	U16	0.25965	0.6666813	0.39	1.0000	-2.08839	2.60769
Athletics	U16	Football	U17	-0.71569	0.2648973	-2.70	0.3843	-1.64866	0.21727
Athletics	U16	Football	U18	-0.62715	0.2695987	-2.33	0.6635	-1.57667	0.32237
Athletics	U16	Multi-sport	U13	1.76849	0.6947634	2.55	0.4980	-0.67846	4.21543
Athletics	U16	Multi-sport	U14	-0.36667	0.3905801	-0.94	1.0000	-1.74228	1.00895
Athletics	U16	Multi-sport	U15	-0.10268	0.3188042	-0.32	1.0000	-1.22550	1.02014
Athletics	U16	Multi-sport	U16	-1.01768	0.4999295	-2.04	0.8492	-2.77842	0.74306
Athletics	U16	Multi-sport	U17	0.37989	0.3698031	1.03	0.9999	-0.92255	1.68233
Athletics	U16	Multi-sport	U18	1.24351	0.5107602	2.43	0.5822	-0.55538	3.04240
Athletics	U17	Athletics	U18	-0.17883	0.2586926	-0.69	1.0000	-1.08994	0.73228
Athletics	U17	Football	U13	0.53127	0.2779487	1.91	0.9053	-0.44766	1.51020
Athletics	U17	Football	U14	0.18321	0.2427583	0.75	1.0000	-0.67178	1.03820
Athletics	U17	Football	U15	0.09673	0.2411629	0.40	1.0000	-0.75264	0.94610
Athletics	U17	Football	U16	0.95474	0.6642545	1.44	0.9938	-1.38475	3.29424
Athletics	U17	Football	U17	-0.02060	0.2587289	-0.08	1.0000	-0.93184	0.89064
Athletics	U17	Football	U18	0.06794	0.2635403	0.26	1.0000	-0.86024	0.99613
Athletics	U17	Multi-sport	U13	2.46358	0.6924350	3.56	0.0447*	0.02483	4.90232
Athletics	U17	Multi-sport	U14	0.32842	0.3864231	0.85	1.0000	-1.03255	1.68940
Athletics	U17	Multi-sport	U15	0.59241	0.3136976	1.89	0.9139	-0.51243	1.69725
Athletics	U17	Multi-sport	U16	-0.32259	0.4966886	-0.65	1.0000	-2.07192	1.42674
Athletics	U17	Multi-sport	U17	1.07499	0.3654099	2.94	0.2369	-0.21198	2.36195
Athletics	U17	Multi-sport	U18	1.93860	0.5075885	3.82	0.0188*	0.15088	3.72632
Athletics	U18	Football	U13	0.71010	0.2985014	2.38	0.6244	-0.34122	1.76142
Athletics	U18	Football	U14	0.36204	0.2660436	1.36	0.9967	-0.57496	1.29904
Athletics	U18	Football	U15	0.27556	0.2645886	1.04	0.9999	-0.65632	1.20743
Athletics	U18	Football	U16	1.13357	0.6731134	1.68	0.9682	-1.23712	3.50427
Athletics	U18	Football	U17	0.15823	0.2806924	0.56	1.0000	-0.83036	1.14683
Athletics	U18	Football	U18	0.24678	0.2851335	0.87	1.0000	-0.75746	1.25101
Athletics	U18	Multi-sport	U13	2.64241	0.7009378	3.77	0.0222*	0.17372	5.11110
Athletics	U18	Multi-sport	U14	0.50726	0.4014604	1.26	0.9986	-0.90668	1.92119
Athletics	U18	Multi-sport	U15	0.77124	0.3320449	2.32	0.6661	-0.39821	1.94070
Athletics	U18	Multi-sport	U16	-0.14376	0.5084753	-0.28	1.0000	-1.93460	1.64709
Athletics	U18	Multi-sport	U17	1.25382	0.3812768	3.29	0.0994	-0.08903	2.59667
Athletics	U18	Multi-sport	U18	2.11743	0.5191278	4.08	0.0073*	0.28908	3.94579
Football	U13	Football	U14	-0.34806	0.2319494	-1.50	0.9900	-1.16498	0.46886
Football	U13	Football	U15	-0.43454	0.2519972	-1.72	0.9604	-1.32207	0.45299
Football	U13	Football	U16	0.42347	0.6684425	0.63	1.0000	-1.93077	2.77772
Football	U13	Football	U17	-0.55187	0.2692804	-2.05	0.8420	-1.50027	0.39653
Football	U13	Football	U18	-0.46333	0.2736955	-1.69	0.9666	-1.42728	0.50063
Football	U13	Multi-sport	U13	1.93231	0.6964696	2.77	0.3355	-0.52064	4.38526
Football	U13	Multi-sport	U14	-0.20284	0.3936070	-0.52	1.0000	-1.58912	1.18343
Football	U13	Multi-sport	U15	0.06114	0.3225055	0.19	1.0000	-1.07472	1.19700
Football	U13	Multi-sport	U16	-0.85386	0.5022979	-1.70	0.9653	-2.62294	0.91523
Football	U13	Multi-sport	U17	0.54372	0.3729987	1.46	0.9927	-0.76998	1.85741
Football	U13	Multi-sport	U18	1.40733	0.5130787	2.74	0.3563	-0.39972	3.21439
Football	U14	Football	U15	-0.08648	0.2098376	-0.41	1.0000	-0.82552	0.65257
Football	U14	Football	U16	0.77154	0.6544613	1.18	0.9994	-1.53346	3.07654
Football	U14	Football	U17	-0.20381	0.2322766	-0.88	1.0000	-1.02188	0.61427
Football	U14	Football	U18	-0.11526	0.2364071	-0.49	1.0000	-0.94788	0.71736
Football	U14	Multi-sport	U13	2.28037	0.6831881	3.34	0.0865	-0.12580	4.68655
Football	U14	Multi-sport	U14	0.14522	0.3695978	0.39	1.0000	-1.15650	1.44694
Football	U14	Multi-sport	U15	0.40921	0.2927215	1.40	0.9954	-0.62175	1.44017
Football	U14	Multi-sport	U16	-0.50579	0.4837140	-1.05	0.9999	-2.20943	1.19784
Football	U14	Multi-sport	U17	0.89178	0.3475689	2.57	0.4827	-0.33235	2.11591
Football	U14	Multi-sport	U18	1.75540	0.4948998	3.55	0.0462*	0.01237	3.49843
Football	U15	Football	U16	0.85802	0.6481493	1.32	0.9976	-1.42475	3.14079
Football	U15	Football	U17	-0.11733	0.2106229	-0.56	1.0000	-0.85914	0.62448
Football	U15	Football	U18	-0.02878	0.2216538	-0.13	1.0000	-0.80944	0.75188
Football	U15	Multi-sport	U13	2.36685	0.6826228	3.47	0.0591	-0.03733	4.77104
Football	U15	Multi-sport	U14	0.23170	0.3685519	0.63	1.0000	-1.06634	1.52973
Football	U15	Multi-sport	U15	0.49568	0.2913998	1.70	0.9651	-0.53062	1.52199
Football	U15	Multi-sport	U16	-0.41931	0.4829153	-0.87	1.0000	-2.12013	1.28151
Football	U15	Multi-sport	U17	0.97826	0.3464564	2.82	0.3044	-0.24195	2.19847
Football	U15	Multi-sport	U18	1.84188	0.4941191	3.73	0.0257*	0.10160	3.58216
Football	U16	Football	U17	-0.97534	0.6529113	-1.49	0.9905	-3.27489	1.32420
Football	U16	Football	U18	-0.88680	0.6614025	-1.34	0.9972	-3.21625	1.44265
Football	U16	Multi-sport	U13	1.50884	0.9214382	1.64	0.9757	-1.73645	4.75413
Football	U16	Multi-sport	U14	-0.62632	0.7203506	-0.87	1.0000	-3.16338	1.91074
Football	U16	Multi-sport	U15	-0.36233	0.6840967	-0.53	1.0000	-2.77171	2.04705
Football	U16	Multi-sport	U16	-1.27733	0.7850361	-1.63	0.9771	-4.04221	1.48755
Football	U16	Multi-sport	U17	0.12024	0.7093001	0.17	1.0000	-2.37790	2.61839
Football	U16	Multi-sport	U18	0.98386	0.7919774	1.24	0.9989	-1.80547	3.77319
Football	U17	Football	U18	0.08854	0.2422806	0.37	1.0000	-0.76476	0.94185
Football	U17	Multi-sport	U13	2.48418	0.6890247	3.61	0.0384*	0.05745	4.91091
Football	U17	Multi-sport	U14	0.34902	0.3802783	0.92	1.0000	-0.99031	1.68836
Football	U17	Multi-sport	U15	0.61301	0.3060963	2.00	0.8657	-0.46505	1.69108
Football	U17	Multi-sport	U16	-0.30199	0.4919230	-0.61	1.0000	-2.03453	1.43056
Football	U17	Multi-sport	U17	1.09559	0.3589055	3.05	0.1833	-0.16847	2.35964
Football	U17	Multi-sport	U18	1.95920	0.5029263	3.90	0.0143*	0.18791	3.73050
Football	U18	Multi-sport	U13	2.39564	0.6908457	3.47	0.0591	-0.03751	4.82878
Football	U18	Multi-sport	U14	0.26048	0.3835681	0.68	1.0000	-1.09044	1.61140
Football	U18	Multi-sport	U15	0.52447	0.3101738	1.69	0.9670	-0.56796	1.61690
Football	U18	Multi-sport	U16	-0.39053	0.4944706	-0.79	1.0000	-2.13205	1.35099
Football	U18	Multi-sport	U17	1.00704	0.3623893	2.78	0.3326	-0.26929	2.28337
Football	U18	Multi-sport	U18	1.87066	0.5054183	3.70	0.0281*	0.09058	3.65073
Multi-sport	U13	Multi-sport	U14	-2.13515	0.7464162	-2.86	0.2822	-4.76402	0.49371
Multi-sport	U13	Multi-sport	U15	-1.87117	0.7114918	-2.63	0.4353	-4.37703	0.63469
Multi-sport	U13	Multi-sport	U16	-2.78617	0.8090203	-3.44	0.0635	-5.63552	0.06319
Multi-sport	U13	Multi-sport	U17	-1.38859	0.7357573	-1.89	0.9143	-3.97992	1.20273
Multi-sport	U13	Multi-sport	U18	-0.52498	0.8157576	-0.64	1.0000	-3.39806	2.34811
Multi-sport	U14	Multi-sport	U15	0.26399	0.4123967	0.64	1.0000	-1.18847	1.71644
Multi-sport	U14	Multi-sport	U16	-0.65101	0.5691634	-1.14	0.9996	-2.65560	1.35357
Multi-sport	U14	Multi-sport	U17	0.74656	0.4595539	1.62	0.9775	-0.87198	2.36510
Multi-sport	U14	Multi-sport	U18	1.61018	0.5790657	2.78	0.3315	-0.42928	3.64964
Multi-sport	U15	Multi-sport	U16	-0.91500	0.5117659	-1.79	0.9454	-2.71743	0.88743
Multi-sport	U15	Multi-sport	U17	0.48257	0.3998759	1.21	0.9992	-0.92578	1.89093
Multi-sport	U15	Multi-sport	U18	1.34619	0.5332920	2.52	0.5140	-0.53205	3.22444
Multi-sport	U16	Multi-sport	U17	1.39757	0.5390543	2.59	0.4627	-0.50097	3.29611
Multi-sport	U16	Multi-sport	U18	2.26119	0.6577946	3.44	0.0647	-0.05555	4.57793
Multi-sport	U17	Multi-sport	U18	0.86362	0.5652598	1.53	0.9879	-1.12722	2.85445
